# Leptomeninges: Anatomy, Mechanisms of Disease and Neuroimaging

**DOI:** 10.3390/neurolint17120203

**Published:** 2025-12-15

**Authors:** Marialuisa Zedde, Rosario Pascarella

**Affiliations:** 1 Neurology Unit, Stroke Unit, Azienda Unità Sanitaria Locale-IRCCS di Reggio Emilia, Viale Risorgimento 80, 42123 Reggio Emilia, Italy; 2 Neuroradiology Unit, Ospedale Santa Maria della Misericordia, AULSS 5 Polesana, 45100 Rovigo, Italy; rosario.pascarella@aulss5.veneto.it

**Keywords:** leptomeninges, arachnoid mater, cerebrospinal fluid (CSF), neuroimaging, MRI, embryology, neurodevelopmental disorders, hydrocephalus, meningeal anatomy

## Abstract

Background: The leptomeninges, comprising the arachnoid and pia mater, serve essential roles in protecting the brain and facilitating cerebrospinal fluid (CSF) circulation. Their significance extends beyond structural support, affecting brain development and function. Methods: This study synthesizes findings from various anatomical, embryological, and neuroimaging research to elucidate the complexities of the leptomeninges. Key methodologies include historical anatomical analysis, contemporary imaging techniques, and examination of pathological states. Results: The review highlights the role of leptomeningeal structures in CSF dynamics, neurovascular interactions, and their involvement in conditions such as hydrocephalus and neurodevelopmental disorders. These insights underscore the leptomeninges’ critical involvement in both normal physiology and disease states. Conclusions: Understanding the intricacies of leptomeningeal anatomy and function is vital for advancing diagnostic and therapeutic approaches in neurodegenerative disorders. This knowledge may facilitate better management strategies in clinical practice, particularly concerning conditions that disrupt CSF flow and brain health.

## 1. Introduction

The meninges serve as a protective covering for the delicate neural tissue of the brain and spinal cord. They also play a crucial role in anchoring the CNS parenchyma to the bony structures of the skull and vertebral column. Additionally, the meninges encompass spaces that facilitate the circulation of cerebrospinal fluid (CSF) around the CNS. In addition to their structural roles, new research indicates that the meninges play an active part in brain development and calvarial formation, potentially serving as a postnatal stem cell niche [1,2,3,4]. Clinically, abnormalities in the meninges have been associated with two neurodevelopmental disorders in humans, i.e., Dandy-Walker malformation and cobblestone lissencephaly, both of which interfere with normal brain function [3]. Despite their important functions, our knowledge regarding the formation, differentiation, and molecular attributes of the meninges is still quite limited.

Galen documented the dura and pia mater in the 2nd century AD [1], but the arachnoid mater was not recognized until the 17th century. In 1664, Gerardus Blasius (1626–1692) identified and named the arachnoid layer, emphasizing its cobweb-like structure. Frederick Ruysch (1638–1731) later confirmed its role as a complete layer surrounding the brain [5,6]. In the 1690s, Humphrey Ridley (1653–1708) introduced the concept of the subarachnoid cistern, detailing various structures such as the cerebellomedullary, quadrigeminal, and olfactory cisterns [6].

The function of the arachnoid membranes in regulating cerebrospinal fluid (CSF) flow became evident in the 19th century. François Magendie (1783–1855) demonstrated in 1842 that CSF in the ventricles communicated freely with the subarachnoid space. Subsequently, in 1869, Axel Key (1832–1901) and Magnus Retzius (1842–1919) illustrated the subarachnoid cisterns and confirmed CSF passage through openings in the arachnoid using dye injection experiments [7].

Gazi Yasargil (1925), a pioneer in microneurosurgery, highlighted the importance of arachnoid membranes for surgical navigation between cisterns, noting their varying degrees of porosity and potential obstruction following subarachnoid hemorrhage [8].

The anatomical significance of the subarachnoid space in radiology dates back to 1919, when Walter Dandy advanced pneumoencephalography, enabling intracranial tumor diagnosis. This method, though invasive, allowed for enhanced imaging of the brain’s structures. In 1956, Bengt Liliequist (1923–2008) observed that air often paused in the interpeduncular cistern during pneumoencephalography, leading to his description of the Liliequist membrane (LM) [9,10]. The LM is typically visible on lateral pneumoencephalograms as a fine line extending from the dorsum sellae to the mammillary bodies. As pneumoencephalography became less common due to the rise of non-invasive imaging techniques like CT and MRI, the routine radiologic visualization of arachnoid membranes also declined, resulting in a diminished understanding of their anatomy within the radiological community. Fortunately, advancements in high-field-strength and higher-resolution MRI have allowed for improved visualization of these structures. However, many contemporary neuroradiologists and current literature still lack awareness of the anatomy and significance of arachnoid membranes, despite their influence on clinical imaging and their potential role in addressing conditions related to abnormal CSF flow. While these membranes are often underappreciated in the radiological field, imaging assessments of them are generally feasible [11,12]. Recognizing these structures is vital due to their numerous clinical and surgical implications, including enhancing preoperative planning, addressing potential endoscopic ventricular shunt failures, and understanding the clinical presentation of suprasellar arachnoid cysts and perimesencephalic hemorrhages [11,12].

The topic of this narrative review is the arachnoid membrane and the subarachnoid space from the embryological, anatomical and pathophysiological perspective, aiming to provide the main background for the most frequent neuroradiological patterns in several diseases. These issues do not allow to be addressed using a systematic approach, including a scoping review. Therefore, a pre-defined protocol for searching and selecting studies was not used, rather allowing a focus on qualitative interpretation. 

## 2. Leptomeninges: Anatomical and Embryological Remarks

In adult mammals, the meninges consist of three distinct layers: the outer dura mater, the middle arachnoid mater, and the innermost pia mater. Each layer is named for its histological features, with “dura” meaning tough and “mater” meaning mother, indicating the dura mater’s robust structure. The term “arachnoid” derives from “arachne,” meaning spider, reflecting its web-like appearance, while “pia” translates to tender, highlighting the delicate nature of the pia mater. The dura mater is also known as pachymeninx (from “pachy,” meaning thick), whereas the arachnoid and pia mater are collectively referred to as leptomeninx (from “lepto,” meaning thin) [1].

The dura mater is a robust, dense collagenous membrane that adheres firmly to the inner surface of the skull. It comprises two main layers: the outer endosteal layer, which acts as the periosteum for the inner skull bone, and the inner meningeal layer, often referred to as the dura mater proper. Typically, these layers are fused together, with exceptions found at the dural venous sinuses, which facilitate the drainage of venous blood from the brain. The dura mater extends into the cranial cavity, giving rise to dural reflections. For example, the falx cerebri separates the two cerebral hemispheres, while the tentorium cerebelli distinguishes the cerebral hemispheres from the cerebellum. Additionally, the dura mater—particularly the endosteal layer—contains a rich supply of blood vessels that primarily nourish the calvaria and also includes lymphatic vessels that assist in draining CSF from the central nervous system [1,13,14,15].

In contrast, the arachnoid mater is a thin, translucent membrane made up of several layers of flattened cells. Beneath this membrane is the arachnoid trabeculae, a spongy connective tissue structure composed of collagen fibers and fibroblasts. The openings within this mesh create the subarachnoid space, where blood vessels and CSF circulate [2].The pia mater, on the other hand, is a delicate membrane consisting of a single layer of cells that closely adheres to the brain’s surface. Blood vessels extend from the subarachnoid space through the pia mater into the brain tissue, with the surrounding connective tissue and perivascular spaces regarded as extensions of the pia mater [2,16].

Just beneath the pia mater lies the pial basement membrane, a sheet of extracellular matrix (ECM) containing various proteins, including laminins, collagen IV, heparan sulfate proteoglycans, and nidogen (also known as entactin) [17]. This pial basement membrane acts as a barrier between the meninges and brain parenchyma, playing a vital role in brain development.

The embryonic development of the meninges has been extensively studied across various species, particularly in mammals such as humans, mice, and rats [1]. During the early stages of embryogenesis, mesenchymal cells begin to envelop the hindbrain as the neural tube closes, gradually extending to the midbrain and forebrain regions [16]. By Carnegie stage 15 in humans, which occurs around the 5th week of gestation, and at approximately embryonic day (E) ~E9.5 in mice, a mesenchymal sheath forms over the developing brain. This structure is referred to as the primary meninx (or primitive meninx), which serves as the foundational layer for the meninges, skull, and scalp [16,18,19]. This primary meninx serves as the foundation for the development of the meninges, skull, and scalp [16]. Additionally, the primary meninx contains a vascular plexus, known as the perineural vascular plexus, located along the brain’s surface. This plexus will develop into the blood vessels that integrate into the meninges and extend into the brain tissue [2]. Early histological identification of the pia mater can be observed in specific brain regions, where it is characterized by cells situated between the vascular plexus and the brain wall [16]. Fibroblasts present within the pia mater are responsible for producing ECM proteins that constitute the pial basement membrane, effectively separating the meninges from the brain parenchyma.

Around embryonic day (E) 10.5 in mice, the primary meninx differentiates into two distinct layers: an outer dense layer and an inner reticular layer. The inner layer is known as the meningeal mesenchyme, although it remains unclear whether it gives rise to all three meninges, including the dura mater. By stage 17 in humans, approximately the 6th week of gestation, and around E13 in mice, the mesenchyme surrounding the brain organizes into well-defined layers. The outermost layer is the dermal layer, followed by a ‘skeletogenic’ layer responsible for skull formation [16].

Recent studies indicate that the mesenchymal cells in this skeletogenic layer, particularly on the apical side of the head, may be more involved in the formation of sutures (soft tissue joints) rather than the bony plates of the calvaria [20,21]. Therefore, designating this layer as the ‘calvarial layer’ [3] is more precise, as it includes precursors for both sutures and bone structures. As development continues, the meningeal primordium begins to divide into the pachymeninx (dura mater) and leptomeninx (which encompasses the arachnoid mater and pia mater) through a structure known as the dural limiting layer. The pachymeninx is composed of fibroblasts arranged longitudinally, whereas the leptomeninx is characterized by a loosely organized meshwork of cells. The dural limiting layer, consisting of condensed cells, is thought to contribute to both the dura mater and the arachnoid mater, with its outer section typically included in the definition of pachymeninx [16].

The differentiation of these meningeal layers occurs in a basal-to-apical progression [22]. During this process, the leptomeninx undergoes cavitation, leading to the formation of the arachnoid trabeculae and the subarachnoid space. Simultaneously, the dura mater accumulates collagen fibers [18], and lymphatic vessels within the dura mater begin to develop during early postnatal life [23].

Initial experiments utilizing quail and chick chimeras have provided insights into the developmental origins of the meninges. These investigations revealed that neural crest-derived cells, originating from the caudal forebrain and midbrain regions, specifically contribute to the meninges associated with the forebrain. In contrast, the meninges of the midbrain and hindbrain are formed by cells derived from the mesoderm [24,25]. It is noteworthy that endothelial cells within the blood vessels of the meninges across all regions are exclusively of mesodermal origin [24]. Histological examinations of human fetuses corroborate these findings, indicating that the cranial meninges develop from a combination of neural crest cells, derived from the ectoderm, and mesodermal cells. Specifically, the prechordal plate and paraxial mesoderm have been identified as significant sources of mesodermal cells contributing to meningeal development [16].

This understanding of the origins of meningeal cells highlights the complex interplay between different embryonic germ layers in the formation of the protective layers surrounding the brain.

### 2.1. Arachnoid Mater

The arachnoid mater is a fragile and avascular layer that lies directly against the dura mater, and it can be easily separated by a potential space referred to as the “subdural space”. This layer is distinct from the pia mater, which is separated from the arachnoid mater by the CSF-filled subarachnoid space. Within the subarachnoid space, delicate filaments called arachnoid trabeculae serve to connect the arachnoid mater to the pia mater.

The arachnoid mater closely follows the contours of the pia mater, covering the entire brain, including the superior surface of the pituitary fossa. It becomes continuous with the spinal arachnoid at the foramen magnum. Unlike the pia mater, the arachnoid bridges over the sulci and fissures of the brain, with the exception of the significant longitudinal fissure that separates the two cerebral hemispheres. It encases blood vessels and nerves as they enter the cranial cavity through the subarachnoid space, merging with the epineurium and adventitia of these structures upon exit.

In the case of the optic nerve, the arachnoid mater and the subarachnoid space surround the nerve as it extends into the orbital cavity, merging with the sclera of the eyeball. Additionally, the arachnoid mater reflects onto the surfaces of blood vessels within the subarachnoid space, primarily adhering to the internal carotid and vertebral arteries.

From an anatomical perspective, the arachnoid mater is thicker in the basal region, particularly between the temporal lobes and at the anterior surface of the pons. As individuals age, the arachnoid layer on the superior surface may become white and opaque, especially near the midline [1].

### 2.2. Subarachnoid Space

The discovery and understanding of CSF have evolved significantly over the centuries. Early descriptions noted the presence of fluid in the ventricles and around the brain, with the identification of CSF credited to Contugno in 1764 and later rediscovered by Magendie in 1825 [26,27]. Rokitansky, in 1844, stated that the arachnoid mater forms a sac that contains this fluid, while Virchow contested the notion of a normal fluid content within the sac in 1856 [28,29].

Key contributions to the understanding of the cerebrospinal fluid (CSF)-filled subarachnoid space and its connection to the ventricles were made by Key and Retzius, who determined that this space encircles the brain and communicates with the ventricles [30,31,32]. In 1800, Bichat proposed that the arachnoid forms a closed sac that connects with the third ventricle, a concept that Magendie later expanded upon regarding the fourth ventricle. In 1855, von Luschka discovered that the fluid within the arachnoid sac is linked to the fourth ventricle and the spinal cord [33,34].

The subarachnoid space, located between the arachnoid and pia mater, varies in thickness and is absent where the brain directly contacts the pia mater. It communicates with the fourth ventricle through the foramen of Magendie and the foramina of Luschka [33]. According to Hodges, this space can be classified into basilar cisterns and channels that traverse the cerebral hemispheres, containing CSF and extending into the sulci [35]. Magendie reported findings in 1828 regarding an opening in the roof of the fourth ventricle, which was later confirmed by Luschka, Key, and Retzius, though it faced some disagreement from other contemporaries [27,30,33,35,36,37,38].

The foramina of Luschka, described by von Luschka in 1855, establish a connection between the fourth ventricle and the subarachnoid space [33]. Within the subarachnoid space, various cisterns exist, including the ambient cistern, which surrounds the brainstem and connects with the pontine and crural cisterns, potentially dividing into supratentorial and infratentorial compartments [8,9,39]. The quadrigeminal cistern, located at the back of the tentorial notch, contains numerous arteries and veins [8,39]. The callosal cistern is situated between the falx cerebri, the cerebral hemispheres, and the corpus callosum, and it is divided into anterior and posterior portions [8,39]. The cerebellomedullary cistern, the largest subarachnoid cistern, is found between the medulla oblongata and the cerebellum, housing several cranial nerves and arteries [8,9,40].

Lu and Zhu [40,41] explored cranial arachnoid membranes, classifying them into convex, basal, and trabecular types, highlighting their significance in delineating cisterns and facilitating surgical exposure.

### 2.3. Arachnoid Membranes

The subarachnoid space is characterized by connective tissue projections lined with leptomeningeal cells, which serve to connect the arachnoid mater to the pia mater. These projections take on various forms, ranging from slender cylinders commonly referred to as “pillars,” “rods,” or “trabeculae,” to elongated, flattened structures known as “septa” [42]. In some regions, these septa are extensive enough to create semicontinuous layers termed “arachnoid membranes” (also called “sub-arachnoid trabecular membranes” or “inner arachnoid membranes”). This network of fine, continuous, sheet-like trabeculae effectively divides the subarachnoid space into compartments, thereby facilitating a more directed flow of CSF.

The trabeculae extend from the deeper layers of the arachnoid mater to the pia mater, encasing small blood vessels and adhering to the surfaces of larger blood vessels within the subarachnoid space. At their attachment sites, trabecular cells connect with the cells on the surface of the pia or the blood vessels. These trabeculae feature a collagen core surrounded by leptomeninges, which are interconnected by desmosomes and gap junctions [43].

According to Yaşargil [8], the trabeculae also attach to nerves located within the subarachnoid space. Neural elements, including nerve endings found in the arachnoid and trabeculae—particularly in the cisterna magna—may transmit information regarding CSF pressure and could play a role in conditions such as cerebral vasospasm. Additionally, fine capillaries have been observed within the trabeculae in rats [8], indicating a potential role in microcirculation within the subarachnoid space. The LM is a crucial component of the trabecular arachnoid and serves as an important anatomical landmark for accessing the sellar and parasellar regions. Numerous authors, including Liliequist [9], Yaşargil [8], Brasil [44], and Vinas [45], describe this membrane as typically single-layered. However, Froelich et al. [12] noted that it can exhibit complexity and variability, sometimes appearing as either a single or double-layered membrane. Matsuno [46] and Rhoton [47] identified two distinct parts: the mesencephalic and diencephalic leaves, while Lu and Zhu [40,41] recognized three leaves: mesencephalic, diencephalic, and a pair of diencephalic–mesencephalic leaves. Wang et al. [48] indicated that this membrane comprises three layers: mesencephalic, diencephalic, and a pair of hypothalamic membranes. Each study provides various descriptions of the LM concerning its morphology, orientation, attachment, classification, and relationship with surrounding structures.

The subarachnoid cisterns, typically named after adjacent brain structures, are generally separated by arachnoid membranes, which help regulate the flow of CSF between different cisterns. The significance of these membranes is well recognized in neurosurgery, as they, along with the subarachnoid cisterns, provide natural pathways for surgical dissection [8].

The embryologic development of the subarachnoid space and its membranes occurs in two distinct phases. The first phase involves the formation of a primitive mesenchymal layer known as the meninx primitiva, or primitive subarachnoid space. This phase begins shortly after the closure of the neural tube, around days 10 to 13 post-conception (approximately the fourth week of pregnancy). During this period, mesenchymal cells migrate from the developing cervical spine into the space between the embryonic epithelium and neuroepithelium of the telencephalon [16,49]. At this stage, the meninx primitiva consists of a gel-like layer of glycosaminoglycans interspersed with widely spaced pluripotent stellate mesenchymal cells and a vascular plexus [2].

In the second phase, fluid-filled cavities or lacunae develop within the gel-like meninx primitiva, gradually enlarging and separating the mesenchymal cells. Where these cavities converge, the cells become compacted, leading to membrane formation. This stage may also give rise to primary or congenital arachnoid cysts due to duplication of the arachnoid membranes or failure of meningeal fusion [50,51]. As mesenchymal cells differentiate on both the inner and outer surfaces of the meninx primitiva, they ultimately give rise to the arachnoid and pia mater, typically completing this process by day 17 [16,49]. Thus, the arachnoid membranes represent the remaining trabeculated structures that connect the two inner layers of the meninges.

Arachnoid septa, trabeculae, and membranes are projections that extend from the arachnoid mater to the pia mater, playing a crucial role in the structural organization of the subarachnoid space. Arachnoid trabeculae consist of collagen bundles coated by leptomeningeal cells, which are interconnected through desmosomes and gap junctions [15]. Within the subarachnoid space, small vessels are encased by these trabeculae, while larger vessels are covered by leptomeningeal cells that maintain continuity with those in the trabeculae.

At the junction where the trabeculae meet the pia mater, their cellular layers merge with the outer layer of the pia mater. In certain regions, dense networks of semicontinuous trabeculae can resemble true membranes [45], effectively partitioning the subarachnoid space into distinct cisterns and providing structural support for the neurovascular structures that traverse these areas. The appearance of the arachnoid membranes shows considerable variability; they can be transparent or semitransparent, membranous or reticulated, porous or intact, and their thickness can differ across various specimens [41]. These membranes are typically named based on their anatomical relationships to surrounding brain structures, with the exception of the eponymous LM.

The microsurgical anatomy of various arachnoid membranes has been extensively documented [41]. However, identifying these membranes can be challenging, even with high-resolution MRI techniques. Their precise attachments and boundaries are described with variability, reflecting both descriptive differences and actual anatomical variability. Notably, abnormally thickened membranes are often observed in regions associated with the interpeduncular and prepontine cisterns, particularly in some patients with communicating hydrocephalus.

The interpeduncular cistern is bordered posteriorly by the midbrain and anteriorly by the two leaves of the LM. The LM originates from the posterior clinoid processes and the dorsum sellae, dividing into two sheets: the diencephalic and mesencephalic leaves. The diencephalic leaf attaches to the diencephalon near the mammillary bodies and separates the chiasmatic cistern from the interpeduncular cistern. The mesencephalic leaf, divided by the oculomotor nerves into medial and lateral portions, separates the interpeduncular cistern from the prepontine cistern below. This portion is typically thinner and less complete, making it less visible on imaging [52,53,54].

The prepontine cistern is a crucial anatomical space in the brain, situated between several key structures. It is separated from the interpeduncular cistern superiorly by the mesencephalic leaf of the LM, which contributes to the compartmentalization of CSF. Inferiorly, the prepontine cistern is bordered by the medial pontomedullary membrane, located at the pontomedullary sulcus.

Laterally, the prepontine cistern is delineated from the cerebellopontine cisterns by paired paramedian anterior pontine membranes. These membranes extend alongside the basilar artery, forming a boundary between the pons and the clivus. As these membranes extend caudally, they tend to thin out and may even disappear in the lower pons, indicating a transition in anatomical structure.

The significance of the cerebellopontine cistern is particularly evident in clinical scenarios, such as subarachnoid hemorrhage, where the hemorrhage is partially confined to this region, underscoring its importance in both anatomy and pathology.

The LM extends posterior-superiorly from the dorsum sellae and the posterior clinoid process. Its diencephalic leaf continues along this trajectory, attaching to or just anterior to the mammillary bodies, effectively separating the interpeduncular cistern from the chiasmatic cistern. The mesencephalic leaf of the LM projects posteriorly and is divided by the oculomotor nerves into medial and lateral segments. The medial segment, which attaches to the distal basilar artery or at the pontomesencephalic junction, serves to separate the interpeduncular cistern from the prepontine cistern. In contrast, the lateral segments connect to the tentorium and mesial temporal lobes, helping to separate the ambient cisterns from the cerebellopontine cisterns.

The anterior pontine membranes run alongside the basilar artery and are positioned medially to the abducens nerves, effectively separating the prepontine cistern from the cerebellopontine cisterns. Additionally, the medial pontomedullary membrane, located at the midline of the pontomedullary sulcus, divides the prepontine cistern from the premedullary cistern. The lateral pontomedullary membranes further separate the cerebellomedullary cisterns from the cerebellopontine cisterns, illustrating the complex anatomical organization of these structures.

CSF flows into the prepontine cistern from both the cerebellomedullary and premedullary cisterns. Within the prepontine cistern, CSF moves superiorly and subsequently enters the interpeduncular cistern. From the interpeduncular cistern, it can flow laterally and anteriorly into the chiasmatic cistern or posteriorly into the ambient cisterns.

### 2.4. The Subarachnoid Lymphatic-like Membrane (SLYM)

Recent studies have suggested the presence of a fourth membrane, based on animal research, which investigates the organization of CSF and immune cell movement within the subarachnoid space surrounding the brains of both mice and humans [55]. There is increasing evidence that CSF may function similarly to a lymphatic system within the central nervous system [56]. Cardiovascular pulsatility promotes CSF inflow through peri-arterial spaces into deeper brain regions [57,58], facilitating CSF exchange with interstitial fluid, aided by glial aquaporin 4 (AQP4) water channels [59]. Various pathways, including perivenous spaces and cranial nerves, assist in clearing fluid and solutes from the neuropil, ultimately directing them to venous circulation via meningeal and cervical lymphatic vessels [13,15]. While CSF reabsorption at the sinuses through arachnoid granulations is recognized, this process has not been thoroughly examined in rodents. Although significant research has focused on CSF flow along the glymphatic-lymphatic pathway, the dynamics of CSF within the extensive subarachnoid space remain inadequately understood [60,61].

In their investigation, the authors utilized in vivo two-photon microscopy to examine meningeal membranes within the somatosensory cortex of Prox1-EGFP+ reporter mice (where Prox1 is a transcription factor guiding lymphatic fate). Below the parallel collagen bundles in the dura, they identified a continuous monolayer of flattened Prox1-EGFP+ cells interspersed with loosely arranged collagen fibers. This newly identified structure, referred to as the SLYM, partitions the subarachnoid space into an outer superficial compartment and an inner deep compartment lining the brain. Quantitative analysis indicated that the thickness of the SLYM was 14.2 ± 0.5 μm, making it thinner than the dura, which measured 21.8 ± 1.3 μm (*n* = 6 mice). The dura’s vasculature is surrounded by collagen fibers, while the SLYM envelops the subarachnoid vessels, demonstrating distinct organizational and dimensional differences between the two vascular systems. The authors hypothesized that the SLYM functions as an impermeable membrane that compartmentalizes the subarachnoid space. To test this hypothesis, Prox1-EGFP+ mice received injections of 1 mm microspheres conjugated to red fluorophores into the outer superficial compartment of the subarachnoid space, alongside an injection of 1 mm microspheres conjugated to blue fluorophores distributed within the inner deep compartment via the cisterna magna. Observations using in vivo two-photon microscopy revealed that the red microspheres were restricted to the outer superficial compartment, while the blue microspheres remained confined to the inner deep compartment. Quantitative analysis confirmed that the 1 mm microspheres did not traverse the SLYM from either compartment. However, many solutes in CSF, such as cytokines and growth factors, are smaller than 1 mm in diameter [62]. When the authors used a smaller tracer, tetramethylrhodamine (TMR)–dextran (3 kDa), administered into the deep inner compartment, they found that it also did not cross the EGFP-expressing SLYM in six mice. In cases where dural damage and CSF leakage occurred, the tracer was observed on both sides of the EGFP+ membrane. Thus, the SLYM appears to separate the subarachnoid space into upper superficial and lower deep compartments for solutes ≥ 3 kDa, acting as a barrier that limits the exchange of most peptides and proteins, including amyloid-β and tau, between these compartments.

While live brain imaging avoids fixation artifacts [63], it does not allow for immunophenotypic characterization of the meningeal membranes. Immunohistochemical analysis revealed that Prox1-EGFP+ cells lined the ventral aspects of the entire brain surface. Immunolabeling indicated that SLYM cells were positive for the lymphatic marker podoplanin (PDPN) [64] but negative for lymphatic vessel endothelial receptor 1 (LYVE1) [65]. The SLYM also labeled for cellular retinoic acid-binding protein 2 (CRABP2), which is typically expressed in dural and arachnoid cells during early development [66]. In contrast, lymphatic vessels in the dura were positive for all classical lymphatic markers—Prox1-EGFP+, PDPN+, LYVE1+, and VEGFR3+—but negative for CRABP2.Notably, analysis of the adult human cerebral cortex showed a CRABP2+/PDPN+ membrane present above the pia mater throughout the subarachnoid space, suggesting that the SLYM also encases the human brain. The authors infer that the SLYM monolayer of Prox1-EGFP+ cells organizes into a membrane structure rather than forming vessels and exhibits a unique set of lymphatic markers. To differentiate the subarachnoid lymphatic-like membrane (SLYM) from arachnoid mater structures, the authors conducted immunolabeling against claudin-11 (CLDN-11), a crucial component of the tight junctions forming the arachnoid barrier cell layer (ABCL) [67]. CLDN-11 was found to be abundantly expressed in the ABCL and in stromal cells of the choroid plexus, while the SLYM was negative for CLDN-11. The ABCL also showed distinct positivity for E-cadherin (E-Cad), in contrast to the SLYM, where surrounding trabecular cells were Prox1-EGFP−/LYVE1−. Pial cells covering the cortical surface exhibited a different immunolabeling profile compared to the SLYM.

The authors concluded that the SLYM constitutes a fourth meningeal layer surrounding both mouse and human brains, displaying lymphatic-like characteristics (Prox1-EGFP+, PDPN+, LYVE1−, CRABP2+, VEGFR3−, CLDN-11−, and E-Cad−). This membrane is phenotypically distinct from the dura, arachnoid, and pia mater. Notably, the SLYM expresses podoplanin (PDPN), a characteristic it shares with mesothelium lining body cavities [25]. PDPN+ cells were also identified in the kidneys and podocytes of adult C57BL/6J mice, as well as in human fetuses, where PDPN+ membranes correspond to the pericardium, pleura, and peritoneum, which envelop developing organs.

The SLYM may represent the brain mesothelium, covering blood vessels in the subarachnoid space. The mesothelium is found in areas where tissues slide against each other and is thought to function as a boundary lubricant, facilitating movement. Physiological pulsations from the cardiovascular system, respiration, and positional changes of the head continuously shift the brain within the cranial cavity. Therefore, the SLYM may, like other mesothelial membranes, reduce friction between the brain and skull during these movements.

## 3. Physiology and Functions

The meninges play a critical role in brain development through several mechanisms, including trophic support and influence on neuron migration. Meninges are believed to provide essential trophic factors that promote the survival of brain cells. Experiments involving the ablation of the neural crest in early chick embryos, which inhibits forebrain meninges formation, revealed significant apoptosis and degeneration of the forebrain neuroepithelium. However, the specific factor(s) responsible for this effect have not yet been identified [68]. The meninges also influence the migration and positioning of neurons by secreting molecules that attract or repel these cells. They express the chemoattractant CXCL12 (chemokine (C-X-C motif) ligand 12, also known as SDF-1), which is activated by the transcription factor FOXC1 [69,70]. Neurons and neural progenitors expressing the receptors CXCR4 and CXCR7 are guided to and retained in the marginal zone of the brain, just beneath the meninges. This regulation affects various cell types, including Cajal-Retzius cells and interneurons in the cerebral cortex, as well as neural progenitors in the dentate gyrus of the forebrain and the cerebellum [69,71,72,73,74,75,76,77]. Conversely, molecules such as BMP4, BMP7, and TGFβ1 secreted by the meninges repel oligodendrocyte precursor cells from the ventral forebrain, directing them toward the cerebral cortex [78]. Additionally, retinoic acid (RA) signaling plays a crucial role in modulating cortical neuron migration. Deletion of an RA-synthesizing enzyme in the meninges has been shown to alter neuron migration and disrupt cortical layering [79,80].

The pial basement membrane serves structurally to regulate neuronal migration and positioning [2,3]. During normal brain development, radial glia extend processes from the ventricular zone to the brain surface. These processes provide a scaffold for the radial migration of neurons, with their endfeet attaching to the pial basement membrane for stability, which halts migrating neurons at the marginal zone. In mouse mutants with defects in the pial basement membrane, premature detachment of radial endfeet resulted in abnormal neuronal distribution in both the cerebral cortex and cerebellum [81].

The meninges also play a role in neurogenesis, the generation of neurons from neural progenitors through asymmetric cell divisions. This was primarily demonstrated in studies involving Foxc1 mutants that lack most of the meninges [82]. In the cerebral cortex of E14.5 Foxc1lacZ/lacZ mutants, an increase in symmetric divisions and a decrease in asymmetric divisions were observed compared to controls, leading to an elongated cortex with fewer differentiated neurons. Interestingly, in utero treatment with RA during E10.5–E13.5 partially rescued the cortical phenotype despite unchanged meningeal defects, indicating that RA from the meninges is critical for cortical neurogenesis [82]. While RA has been shown to be sufficient for promoting neurogenesis in the absence of meninges, its necessity during normal development alongside the meninges remains a topic of debate [79,83]. Beyond the cerebral cortex, the meninges also influence neurogenesis in the embryonic dentate gyrus, with BMP7 identified as a key factor in this process [84]. In the cerebellum, CXCL12 secreted by the meninges inhibits neuronal differentiation while promoting proliferation [85].

The meninges play a crucial role in the development of brain blood vessels. In Foxc1ch/ch and Foxc1lacZ/lacZ mutants, blood vessels in the cerebral cortex exhibited increased diameters but decreased densities at E14.5 [86]. This phenotype is attributed to the absence of the meninges and the resulting loss of retinoic acid (RA). Similarly, inactivation of an RA synthesis enzyme resulted in cerebrovascular defects similar to those observed in Foxc1 mutations [87]. In this context, RA signaling has dual roles in modulating the WNT/β-catenin pathway in endothelial cells, supporting normal vascular growth while preventing excessive expansion [87].

A recent study specifically investigated cerebral vein development, demonstrating that TWIST1-controlled production of BMP2 and BMP4 from the endosteal dura mater and calvarial bone is essential for the growth and remodeling of these veins [88].The meninges also influence the formation of the corpus callosum, the thick bundle of axons connecting the two cerebral hemispheres along the dorsal midline of the brain. BMP7 produced by the meninges inhibits the outgrowth of callosal axons, while WNT3 from neurons counteracts this effect, facilitating corpus callosum development [89].

Additionally, the meninges serve as a niche for neural stem cells, as reviewed by Decimo et al. [2]. A population of cells expressing Nestin, a marker for stem/progenitor cells, has been identified in the leptomeninges of rats from late fetal stages into adulthood [90,91]. Lineage-tracing studies confirmed that these cells act as stem cells for cortical neurons [92]. The meninges’ role as a stem cell niche is thought to be associated with the presence of fractones, specialized extracellular matrix structures rich in growth factors [90].

These findings highlight the meninges’ multifaceted contributions to brain development and function.

The significant influence of the meninges on brain development links defects in these structures to various neurodevelopmental disorders in humans. This condition is characterized by a smooth cerebral cortex lacking the typical ridges and grooves, instead presenting small bumps [93,94]. It occurs when the pial basement membrane’s integrity is compromised, leading to excessive neuronal migration into the meningeal layers. Mutations associated with cobblestone lissencephaly have primarily been identified in genes related to the ECM [93,94]. This disorder is characterized by cerebellar hypoplasia and hydrocephalus (the accumulation of cerebrospinal fluid within the brain) [95]. It has been linked to the FOXC1 gene as one of the associated genes in humans. Research in mice indicates that Foxc1 is expressed in the meninges rather than in the brain [96], suggesting that Dandy-Walker malformation may arise from defects in the meninges. Although the specific meningeal phenotype is often unclear in human patients, imaging studies of individuals with FOXC1 mutations have shown evidence of meningeal deficiencies [96].

These findings highlight the critical role of the meninges in normal brain development and how their defects can lead to severe neurodevelopmental disorders.

### 3.1. Perivascular Subarachnoid Space

For centuries, the brain has been understood to be protected by three meningeal layers: the dura mater, arachnoid mater, and pia mater [97]. However, the discovery of meningeal lymphatic vessels in 2015, which drain CSF to extracranial lymph nodes, has revived interest in the functions of the meninges, particularly their role in lymphatic removal of metabolic waste (e.g., amyloid-β and tau peptides) [13,15,98,99] and immune responses [99,100,101]. A recent study identified a fourth layer, the SLYM, in rodents, which separates the subarachnoid space into outer and inner compartments [55].Human studies have also investigated the anatomical structure of the subarachnoid space, which contains arachnoid trabeculae and membranes that form distinct cisterns [102]. However, the impact of these structures on CSF flow remains poorly understood. Current revisions of CSF flow physiology could significantly enhance our understanding of its role in brain clearance and as a medium for neuroimmune interactions at the meninges [60].

An intriguing study aimed to investigate early CSF tracer propagation in the human subarachnoid space using MRI. The researchers analyzed the time-dependent movement of a tracer to gain insights into CSF dynamics and its correlation with brain tracer enhancement and intracranial pressure (ICP) measurements over a 2–3 h period [103]. Perivascular (periarterial) transport within the subarachnoid space is critical for enhancing CSF and intrathecal drug distribution to brain tissue. This transport was found to be impeded by abnormal intracranial pulsations observed during ICP monitoring, indicating a compromised intracranial pressure-volume reserve capacity. Notably, the dementia subtype idiopathic normal pressure hydrocephalus (iNPH) exhibited enlarged perivascular subarachnoid spaces and impaired tracer transport.

In the study, MRI scans of 75 subjects following the administration of the tracer (gadobutrol, 0.5 mmol) at the lumbar level revealed that it took an average of 13.8 ± 6.3 min for the tracer to reach the foramen magnum. Intracranially, the tracer moved freely within the basal cisterns of the subarachnoid space without barriers, consistently progressing through the sulci near large arteries in a downstream manner. Initially, it formed a temporary circumferential layer around these arteries, followed by broader tracer enrichment in the surrounding subarachnoid space. This periarterial tracer enrichment pattern was documented in several arteries, including the ACA, MCA, and PCA. In contrast, perivenous enhancement was observed less frequently, suggesting that periarterial transport is the primary pattern of tracer propagation. The study indicates a functional compartmentalization of the human subarachnoid space around arteries [103].

The time series data showed that tracer enrichment along arteries followed a time-dependent pattern, with delays noted in distal regions. Additionally, the enrichment of brain tissue was contingent on prior enrichment in the subarachnoid space. The intrathecal tracer accumulated within the periarterial space surrounding the basal cisterns, indicating an unrestricted pathway towards the subarachnoid space of major arteries at the brain’s surface. However, no compartmentalization was observed along the vertebral and basilar arteries. An examination of the relationship between age and tracer transport revealed that delays in tracer appearance increased with age and reduced intracranial compliance. These findings highlight the complexities of CSF dynamics in the human brain and underscore the meninges’ critical role in facilitating CSF transport and clearance.

Patients with iNPH exhibit specific alterations in the dynamics of CSF transport, which have significant implications for brain health. These patients show enlarged periarterial subarachnoid spaces and delayed appearances of tracers compared to control subjects. This underscores the importance of perivascular transport in maintaining brain health and the potential consequences of compromised CSF dynamics in neurodegenerative diseases. The enlarged periarterial subarachnoid spaces in iNPH cases correlate with a decreased rate of perivascular tracer transport.

Furthermore, tracer enrichment in the cerebral cortex was significantly lower at the 2 h mark, particularly affecting the frontal and temporal cortices. The study noted differences in the initial appearance of the tracer in branches of the anterior cerebral artery (ACA) and middle cerebral artery (MCA) among the groups. Specifically, variations were observed in branches such as A1, A2 of the ACA, and the pericallosal artery, as well as M1, M2, and M3 of the MCA. These findings provide in vivo evidence of a compartmentalized human subarachnoid space, characterized by a semipermeable perivascular membrane and a periarterial component that facilitates the antegrade transport of solutes along the anterior, middle, and posterior cerebral arteries toward the cortex. The degree of tracer enrichment in the cerebral cortex is directly correlated with the extent of periarterial tracer enrichment.

Notably, periarterial transport decreases when there is reduced intracranial pressure-volume reserve capacity, which is impaired in conditions like iNPH. The circumferential concentration of the tracer around arteries suggests the presence of a barrier that precedes broader enrichment in the surrounding subarachnoid space, indicating a semi-permeable nature. However, the exact identification of this membrane is limited due to the resolution constraints of MRI. These findings underscore the importance of understanding CSF dynamics and perivascular transport mechanisms, particularly in the context of neurological disorders such as iNPH, where altered CSF flow may significantly impact brain function and health.

Historically, the anatomy of the arachnoid has garnered significant attention from neurosurgeons, with notable contributions from Yasargil [8] and Rhoton [47]. One area of interest is the LM, which is recognized for its potential impact on CSF flow. Although initially described in the 19th century, its functional implications remain poorly understood. Recent findings indicate that a tracer passed freely through the LM, flowing towards perivascular spaces without restriction. However, evidence for a perivascular arachnoid barrier surrounding the vertebral and basilar arteries is lacking, although it cannot be completely ruled out.

Previous studies have shown that the leptomeninges cover arteries in the subarachnoid space [43,104], yet their functional role is still unclear. Recent investigations into the glymphatic system have examined perivascular tracer transport, but not specifically in larger arteries within the subarachnoid space.

It is suggested that the periarterial membrane may parallel the pial sheath described by Zhang et al. [104], which speculated on its role in interstitial fluid drainage. However, current observations indicate that the transport is primarily antegrade along the arteries.

The enhancement around larger veins was minimal, leading to speculation that periarterial propagation of CSF tracer is more significant than that along veins. The findings regarding the recently identified fourth meningeal layer, SLYM [55], complicate the interpretation. While SLYM is suggested to separate the subarachnoid space into outer and inner layers, the current findings indicate that this dichotomy may not apply to arteries within the inner subarachnoid space.

The perivascular subarachnoid space exists around larger arteries at the gyrencephalic brain surface, defined by a semipermeable membrane that allows for directional transport of solutes along the arterial tree. This study indicates that perivascular transport is sensitive to pulsatile intracranial pressure and may be impacted by age and underlying diseases such as iNPH. iNPH shows morphological changes in periarterial spaces and impaired glymphatic tracer clearance, highlighting the importance of understanding these dynamics in relation to brain health.

### 3.2. Meningeal Lymphatics of the Brain: Clearance and Immune Surveillance

For a long time, the CNS was thought to lack a lymphatic system, leading to questions about how the brain clears metabolic waste and maintains fluid homeostasis. Recent research has revealed a complex network of meningeal lymphatic vessels and the glymphatic system, both of which facilitate the drainage of CSF and ISF from the brain. These findings have significant implications for understanding neurodegenerative diseases, such as Alzheimer’s disease, where the accumulation of amyloid-beta and other toxic substances is observed [105].

The glymphatic system is a glia-dependent network of perivascular channels that promotes the exchange of CSF and ISF, which is essential for nutrient delivery and waste removal. According to Iliff et al. [56], CSF flows into the brain’s perivascular spaces, mixing with ISF to facilitate the removal of waste products. This process heavily depends on aquaporin-4 (AQP4) water channels located on the astrocytic endfeet surrounding blood vessels. The authors emphasize the importance of the glymphatic system in maintaining brain health and its potential role in the pathogenesis of neurodegenerative diseases [105].

Dynamic contrast-enhanced MRI has emerged as a powerful tool for visualizing the glymphatic system. Iliff et al. [56] were among the first to use this technique to demonstrate CSF-ISF exchange in live animal models, showing that CSF enters the perivascular spaces and is crucial for clearing metabolic waste from the CNS.

Subsequent studies have utilized MRI to assess glymphatic function in human subjects, providing insights into how factors such as sleep and body posture can influence fluid dynamics in the brain [105].

The identification of meningeal lymphatic vessels has transformed our comprehension of the drainage pathways in the brain. It was previously thought that the CNS lacked lymphatic vessels. However, in 2015, two independent research groups, Aspelund et al. [13] and Louveau et al. [15,106,107,108], confirmed the existence of these vessels in mice through both molecular and functional analyses. These lymphatic vessels play a vital role in the drainage of CSF and ISF from the CNS to the deep cervical lymph nodes, thus supporting the immune response [105].

There is a functional relationship between the glymphatic system and meningeal lymphatics. Studies, including those by Louveau et al. [15,106,107,108] and Da Mesquita et al. [109], have shown that the efficiency of CSF drainage is affected by the health and functionality of both systems. Impairment in one can lead to issues in the other, underscoring the importance of this interconnectedness for maintaining CNS health.

AQP4 channels play a pivotal role in the glymphatic system. Research by Iliff et al. [110] demonstrated that the deletion of AQP4 in mice led to significant impairments in both CSF influx and the clearance of amyloid-beta, suggesting that AQP4 is essential for facilitating brain drainage and preventing the accumulation of neurotoxic substances. The authors stress the importance of AQP4 in the context of neurodegenerative diseases, where its dysfunction could exacerbate pathology [105].

The mechanisms by which CSF is removed from the brain include drainage through arachnoid granulations and lymphatic pathways. The work of Eide and Ringstad [111] has shown that these pathways are vital in clearing solutes from the brain. Various studies highlight the need for understanding these outflow routes, especially in the context of diseases like hydrocephalus and Alzheimer’s disease, where drainage mechanisms may be compromised [105].

Meningeal lymphatic vessels have been identified along the brain’s dural sinuses and cranial nerves, highlighting their significant anatomical location for understanding how the CNS drains interstitial fluid and interacts with the immune system. Recent MRI studies have confirmed the presence of these vessels in humans, suggesting a more considerable role for meningeal lymphatics in cerebrospinal fluid (CSF) drainage than previously recognized [105].

Age-related changes can impair the effectiveness of both the glymphatic and meningeal lymphatic systems. Research indicates that these changes can reduce the drainage of toxic substances, such as amyloid-beta, which is linked to neurodegenerative conditions. Studies by Kress et al. [112] have shown that aging significantly impairs the efficiency of the glymphatic system, emphasizing the need for further research into therapeutic strategies aimed at enhancing lymphatic drainage [105].

The identification of meningeal lymphatics and the glymphatic system challenges the traditional notion of CNS immune privilege. The presence of immune cells within these pathways suggests they play a role in immune surveillance, with evidence indicating that antigen-presenting cells can migrate from the CNS to cervical lymph nodes via the glymphatic system. This raises new questions about the interactions between the immune system and the CNS [105].

The brain’s lymphatic drainage system, which includes the glymphatic system, meningeal lymphatics, and associated drainage pathways, is essential for waste clearance and maintaining fluid homeostasis. Despite recent advancements in understanding these systems, many questions remain regarding their dynamics and interactions, particularly regarding neurological diseases.

## 4. Neuroimaging of the Subarachnoid Space and Biological Mechanisms of Disease

The arachnoid membranes were initially identified using radiological methods during pneumoencephalography. However, with the advent of CT and MRI as standard imaging techniques, pneumoencephalography largely became obsolete by the 1970s [113]. Early CT and MRI technologies had limitations in spatial resolution, making it challenging to visualize the arachnoid membranes.

Recent advancements in high-resolution MRI techniques have enhanced the visualization of arachnoid membranes in routine imaging. Techniques such as True Fast Imaging with Steady-State Precession (CISS) from Siemens, FIESTA and FIESTA-C from GE Healthcare, Balanced Fast Field Echo from Philips Healthcare, Balanced SARGE and phase-balanced SARGE from Hitachi, and True Steady-State Free Precession from Toshiba have all facilitated improved detection of these membranes [102].

When visualized, normal arachnoid membranes appear as delicate T2-hypointense lines crossing the CSF in the subarachnoid space. Due to their thin structure, particularly in healthy individuals, many normal membranes remain elusive and challenging to detect, even with high-resolution, high-field-strength scans. The presence of arachnoid membranes is often inferred from the containment and separation of CSF flow voids and artifacts observed in adjacent subarachnoid cisterns. In cases of subarachnoid hemorrhage, inflammation, or tumors, the arachnoid membranes may thicken, making them easier to identify, especially with specialized high-resolution imaging.

The LM is defined as a slender structure (≤1 mm) that typically has a thickness less than that of the tuber cinereum, situated beneath the floor of the third ventricle. It extends anteriorly from the dorsum sellae to the mammillary bodies and is regarded as a remnant of the primary tentorium. The LM is composed of either a single or double layer of arachnoid tissue and is segmented into three distinct parts:

- Sellar Segment: it is the most frequently identified in imaging studies.
- Diencephalic Segment: it is much less commonly identifiable.
- Mesencephalic Segment: it is often incomplete, thinner, and has a fenestration for the passage of the basilar artery.

Numerous documented cases show the membrane demonstrating lateral insertions into or near the oculomotor nerves, typically extending into the adjacent arachnoid sheaths.

**Figure 1:**
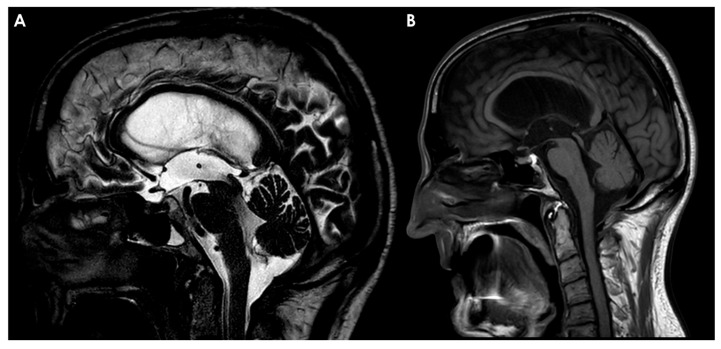
Sagittal MRI (1.5 T) in a 70-year-old patient with hydrocephalus, showing in T2 Drive (**A**) and in T1W (**B**) sequences the sellar segment of the LM.

The posterior anchoring of the LM remains a subject of debate, particularly regarding its potential retromammillary or premammillary insertions [11,12]. An example is provided in Figure 1.

The LM has a relevant role in the brain anatomy, particularly due to its diencephalic component, which serves to separate the interpeduncular cistern from the chiasmatic cistern. Notably, complete blockage of this membrane has been observed in approximately 10–30% of cases, indicating its potential clinical significance and the need for further investigation.

The thin structure of the LM can be optimally evaluated using specific imaging sequences that provide high contrast and anatomical resolution. Among these, the CISS sequences are currently the preferred method for various applications, including:

- Evaluation of cranial nerves
- Examination of cysts and cystic masses
- Diagnosis of neurocysticercosis
- Assessment of hydrocephalus

The CISS sequence is part of the family of fast gradient echo (GRE) sequences and is known by various names depending on the manufacturer:

- FIESTA: Fast Imaging Employing Steady State Acquisition (General Electric)
- FISP: True Fast Imaging with Steady-State Precession (Siemens)
- Balanced FFE: Balanced Fast Field Echo (Philips)
- SSFP: True Steady-State Free Precession (Toshiba) [114].

### 4.1. CSF Flow

The subarachnoid space, which is filled with CSF and contains trabeculated arachnoid membranes, is essential for several critical functions, including nutrient transport, waste elimination, buoyancy (which reduces the effective weight of the brain from approximately 1500 g to around 50 g) [102], and shock absorption. CSF is mainly produced by the choroid plexus located within the ventricles, at an approximate rate of 20 mL/h.

In adults, the total volume of CSF is roughly 130 mL, distributed as follows:

- Ventricles: approximately 30 mL
- Subarachnoid space around the brain: around 25 mL
- Subarachnoid space around the spinal cord: approximately 75 mL [115].

The conventional perspective describes the movement of CSF originating from the choroid plexus and traversing the ventricles in a craniocaudal direction. It exits via the foramina of Luschka and Magendie into the subarachnoid space. Once within the subarachnoid space, CSF moves through the basal cisterns, flows over the convexity of the brain, and progresses along the spinal cord, where it is absorbed into the blood of the cerebral venous sinuses through arachnoid villi [116].

This flow is believed to be driven by hydrostatic pressure gradients from the choroid plexus to the arachnoid villi, along with pulsations from the choroid plexus and contributions from respiratory and cardiac rhythms, resulting in a pulsatile bidirectional flow [117]. Additional influencing factors include posture, jugular venous pressure, physical activity, and the time of day [116].

Recent studies have challenged the traditional view of CSF as merely a bulk flow system. Research has revealed a more complex, multi-directional system involving continuous fluid exchange at the blood–brain barrier and the CSF/interstitial fluid interface [115,118,119,120]. The drainage and absorption of CSF are more intricate, incorporating pathways to cervical and spinal lymph nodes via the cribriform plate and spinal canal nerve roots [115]. Evidence points to the presence of a functional cerebral lymphatic or glymphatic system, where CSF enters the Virchow-Robin perivascular spaces surrounding arteries and arterioles in the subarachnoid space. These vessels are lined by the glia limitans, a delicate layer of astrocytic foot processes enriched with aquaporin-4 (AQP4) water channels. The movement of CSF through these channels occurs via convective flow, facilitating the transport of substances through bulk flow, typically down a pressure gradient [121]. Waste products, including amyloid, dissolve in the CSF before exiting through AQP4 channels in the Virchow-Robin spaces that line the venules and veins [115,120,122].

Despite recent discoveries, a substantial volume of CSF continues to traverse from the ventricles through the basal cisterns and across the convexities of the brain, influenced by the anatomical configuration of the arachnoid membranes [123].

After exiting the fourth ventricle through the foramina of Luschka and entering the cerebellopontine angle, CSF flows into the prepontine cistern via openings in the anterior pontine membranes. It then ascends into the interpeduncular cistern through apertures in the medial part of the mesencephalic leaf of the LM and the medial pontomesencephalic membrane. From the interpeduncular cistern, CSF can either flow ventrally through pores in the diencephalic leaf into the chiasmatic cistern or dorsally around the cerebral peduncles into the ambient cisterns. The ventral movement of CSF from the interpeduncular cistern is somewhat constrained by the diencephalic leaf.

CSF absorption occurs via arachnoid villi/granulations in the venous sinuses, across the cribriform plate in the anterior cranial fossa, at the spinal nerve roots.

Table 1 illustrates the main features of CSF flow.

#### 4.1.1. Hydrocephalus

Arachnoid membranes can undergo thickening due to inflammation or hemorrhage, leading to the occlusion of apertures within these membranes. This can significantly impede CSF flow within the prepontine and interpeduncular cisterns, potentially resulting in hydrocephalus. Thickened arachnoid membranes can displace and alter CSF flow dynamics, as well as be tethered by other membranes, obstructing normal circulation. The resultant condition is often classified as tetraventricular hydrocephalus, typically categorized as communicating hydrocephalus. However, a more precise term would be extraventricular intracisternally obstructive hydrocephalus [124].

These obstructive membranes are usually not visible on conventional MRI. However, they can be detected using higher-resolution imaging sequences such as the 3D-CISS sequence [125,126].

In a study conducted by Laitt et al. [125], “complex membranes” in the basal cisterns were identified in 18 out of 43 patients with hydrocephalus, with findings later confirmed through surgery in those undergoing endoscopic third ventriculostomy (ETV). In patients for whom ETV procedures were unsuccessful, a notable absence of flow voids in the basal cisterns—particularly in the prepontine cistern—was frequently observed, which was attributed to the obstruction of CSF flow caused by these membranes.

Dinçer et al. [126] examined 134 patients with hydrocephalus and found cisternal membranes leading to obstruction in 28 cases, mostly located in the prepontine and interpeduncular cisterns. Seventeen of these cases were surgically validated during ETV, with some membranes being fenestrated during the procedure. For patients with tetraventricular hydrocephalus, the identification of obstructive arachnoid membranes is critical. These individuals, who might otherwise be candidates for shunt insertion, can instead be considered for ETV. Neurosurgeons have the option to fenestrate these thickened membranes during ETV, potentially increasing the success rate of the procedure. iNPH is a neurological condition characterized by gait disturbances, urinary incontinence, dementia. Imaging findings typically reveal enlarged ventricles with normal CSF pressure [127]. Understanding the role of arachnoid membranes in CSF dynamics is essential for effectively diagnosing and treating conditions like iNPH, where CSF flow alterations can significantly impact patient outcomes. Recent studies indicate that impaired clearance of toxic metabolites from the brain via CSF plays a critical role in dementia and neurodegeneration. A recent study investigated MRI biomarkers of CSF tracer dynamics in iNPH patients compared to a reference cohort, aiming to improve understanding of iNPH pathophysiology and its overlap with Alzheimer’s disease [128]. This study involved multi-phase MRI conducted on 34 iNPH patients and 17 reference subjects, all of whom received intrathecal administration of gadobutrol as a CSF tracer. The assessed MRI biomarkers included ventricular reflux grades and molecular clearance rates, compared with conventional anatomical MRI biomarkers (such as Evans’ index, callosal angle, and indicators of disproportionate enlarged subarachnoid space hydrocephalus) and neurodegeneration markers (including Scheltens’ medial temporal atrophy scores, Fazekas scores, and entorhinal cortex thickness). The imaging biomarkers were also correlated with a pulsatile intracranial pressure (ICP) score indicative of intracranial compliance. The study was ethically approved, and all participants provided informed consent.

All iNPH patients exhibited some degree of ventricular reflux following gadobutrol administration, with grades 3–4 observed in 91% of the iNPH cohort compared to only 12% in the reference group. The one-night and two-night clearance of CSF tracer from the cisterna magna was significantly delayed in iNPH patients. The MRI biomarkers of CSF space anatomy and neurodegeneration showed significant differences between iNPH patients and reference subjects, with the iNPH cohort displaying higher scores for both anatomical and neurodegenerative indicators. Notably, ventricular reflux correlated positively with the Evans index and negatively with callosal angle and entorhinal cortex thickness. Logistic regression analysis indicated that imaging biomarkers of CSF tracer dynamics, in conjunction with conventional MRI biomarkers, effectively differentiated iNPH patients from reference subjects. The highest diagnostic ability was noted for the Evans index, ventricular reflux, and one-night clearance of CSF tracer, with area under the receiver operating characteristic curve values exceeding 0.85. These findings suggest that MRI biomarkers of CSF tracer dynamics provide valuable insights into the pathophysiology of iNPH. The high degree of ventricular reflux and delayed CSF tracer clearance in iNPH patients indicate impairment in glymphatic and meningeal lymphatic clearance mechanisms. This impairment is hypothesized to contribute to the accumulation of neurotoxic metabolites, including amyloid-beta and tau, which are also observed in Alzheimer’s disease. The study underscores the potential of MRI biomarkers in assessing CSF dynamics and their role in differentiating pathologies associated with dementia. An enhanced understanding of the relationship between CSF clearance dynamics and neurodegeneration may lead to improved diagnostic strategies and treatment options for patients with iNPH and other forms of dementia.

Disproportionately enlarged subarachnoid space hydrocephalus (DESH) has emerged as a radiological marker with potential prognostic value in iNPH [129]. DESH is characterized by uneven CSF distribution, leading to enlarged ventricles and tight high convexities in the brain. Previous studies have yielded conflicting results regarding the utility of DESH in predicting treatment response, highlighting the need for a systematic review and meta-analysis to clarify its prevalence and diagnostic performance [130]. In total, 812 patients were included in the analysis [131,132]. The meta-analysis revealed a pooled prevalence of DESH in iNPH patients of 44% (95% CI, 34–54%). It was noted that the prevalence of DESH was higher in studies adhering to the second edition of the Japanese guidelines for iNPH management compared to those following international guidelines [133], although this difference was not statistically significant. Regarding diagnostic performance, the pooled sensitivity and specificity of DESH for predicting treatment response were 59% (95% CI, 38–77%) and 66% (95% CI, 57–74%), respectively, with an AUC of 0.67 (95% CI, 0.63–0.71). This indicates a relatively low diagnostic utility for DESH in predicting treatment response. The findings suggest that DESH has a moderate prevalence in iNPH but limited ability to predict outcomes post-surgery. The authors note that previous studies reporting high predictive values for DESH may have been biased due to methodological limitations, such as the absence of control groups or reliance on subjective assessments. This study emphasizes the importance of refining diagnostic criteria and methodologies for evaluating DESH and suggests that better-defined imaging protocols could enhance patient selection for ventriculoperitoneal (VP) shunt surgery. Overall, this meta-analysis demonstrates that while DESH is relatively common in iNPH, its diagnostic performance regarding treatment response prediction is poor.

#### 4.1.2. Endoscopic Third Ventriculostomy

ETV is a surgical procedure aiming to treat non-communicating hydrocephalus by creating a bypass for CSF from the third ventricle to the basal cisterns. Surgical approach typically involves:

- Inserting an endoscope through the brain parenchyma, usually via the right frontal lobe.
- Navigating the endoscope through the foramen of Monro into the third ventricle.
- Creating a stoma at the floor of the third ventricle, allowing CSF to flow directly into the interpeduncular cistern [134].

The diencephalic leaf of the LM is critical for the success of the ETV procedure. Buxton et al. [135] highlighted that this leaf can obstruct CSF flow from the third ventricle to the interpeduncular and prepontine cisterns, making fenestration essential for achieving favorable outcomes during ETV. High-resolution T2-weighted sequences are particularly effective for visualizing CSF flow voids from the third ventricle to the interpeduncular cistern, thereby assisting in surgical planning.

A thorough preoperative evaluation of local anatomy is essential. This includes a detailed investigation and analysis of the arachnoid membranes, which can provide valuable insights for surgical planning and help anticipate potential complications.

After the procedure, CSF flow through the stoma can be visualized and characterized by intense flow artifacts (flow void). Techniques such as phase contrast imaging can be utilized to analyze the pulsatility of CSF flow and measure stroke volume, providing insight into the success of the procedure.

It is important to note that monitoring ventricular dimensions post-procedure is not a reliable parameter for assessing the patency of the stoma. This means that while ventricular size may change, it does not accurately reflect the effectiveness of the ETV in restoring normal CSF flow.

ETV is a critical intervention for managing non-communicating hydrocephalus, particularly when addressing obstructions caused by the diencephalic leaf of the LM. Thorough preoperative evaluations and advanced imaging techniques are essential for optimizing surgical outcomes and ensuring effective CSF drainage. Understanding the dynamics of CSF flow and the anatomy involved is vital for neurosurgeons to enhance the success rates of ETV procedures.

### 4.2. Subarachnoid Hemorrhage

**Figure 2:**
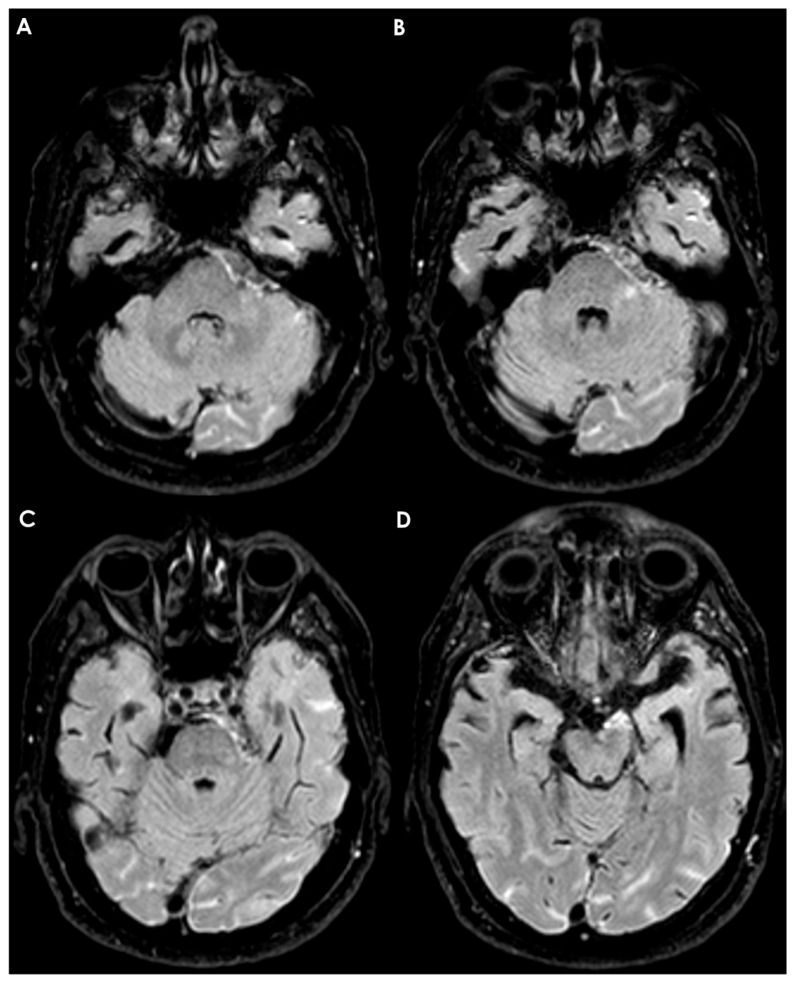
Axial 2D Fluid Attenuated Inversion Recovery (FLAIR) MRI (1.5 T scanner) in ascending slices from the pons to the midbrain (from (**A**–**D**)) showing a compartimentalized irregularly hyperintense signal in the left cerebellopontine cistern, corresponding with subarachnoid bleeding, associated with diffuse sulcal hyperintensities coming from the same source. The patient was a 64-year-old man with a spontaneous subarachnoid bleeding and a negative catheter angiography.

Arachnoid membranes are crucial for separating the cisterns of the brain, and their presence can hinder the spread of blood to adjacent cisterns, similar to how CSF flow might be obstructed. In cases of aneurysmal subarachnoid hemorrhage, the location of the ruptured aneurysm can often be inferred by examining the distribution of blood on the initial unenhanced CT scan. Non-aneurysmal perimesencephalic subarachnoid hemorrhage typically localizes to the interpeduncular, ambient, and prepontine cisterns, without extending into the chiasmatic cistern. This localization is likely influenced by the presence of the diencephalic leaf of the LM [136]. See an example in Figure 2.

The extent of perimesencephalic subarachnoid hemorrhage can vary based on anatomical differences in the arachnoid membranes and the volume of extravasated blood. The concept of the subarachnoid space dates back to the 17th century when British physician Humphrey Ridley first described the basal cisterns, including the cerebellomedullary, quadrigeminal, and olfactory cisterns. He provided evidence of the arachnoid membrane as a distinct meningeal layer, detailing how this membrane enveloped various cerebral vessels and intracranial nerves [6,137].

Historically, it was believed that the subarachnoid space was a continuous area filled with CSF [102]. However, recent studies have challenged this perspective. A study by Mollgard et al. revealed the presence of a mesothelial layer that divides the subarachnoid space into two distinct compartments. This layer, referred to as the SLYM, acts as a semipermeable barrier, limiting the passage of molecules larger than 3 kilodaltons and housing immune cells involved in innate defense mechanisms.

The SLYM was primarily studied in murine models, where its anatomical and functional characteristics were thoroughly characterized. While the study suggests that similar anatomical features may exist in humans, these remain largely unexplored [55].

The role of arachnoid membranes in both CSF dynamics and hemorrhagic events is significant. Understanding how these membranes influence blood distribution and CSF flow is critical for diagnosing and managing conditions such as subarachnoid hemorrhage. Furthermore, the discovery of the SLYM presents an exciting avenue for future research, potentially revealing new insights into the anatomical and functional organization of the subarachnoid space in humans. Further studies are needed to explore the implications of the SLYM for brain health and disease. Recent research has focused on investigating the SLYM in human patients, particularly in the context of pathological conditions such as subarachnoid hemorrhage (SAH) [138].

The fragility of the SLYM poses significant challenges for histopathological assessments. During the dissection of cadaveric specimens, the SLYM is prone to fragmentation, which contributes to its elusive nature and explains why it has remained largely undiscovered until now.

In vivo imaging offers an alternative method for studying the SLYM; however, the use of iodinated contrast or gadolinium-based contrast agents is not feasible due to their small molecular size [139,140]. This size limitation hampers the effective assessment of the SLYM using these commonly employed imaging agents.

**Figure 3:**
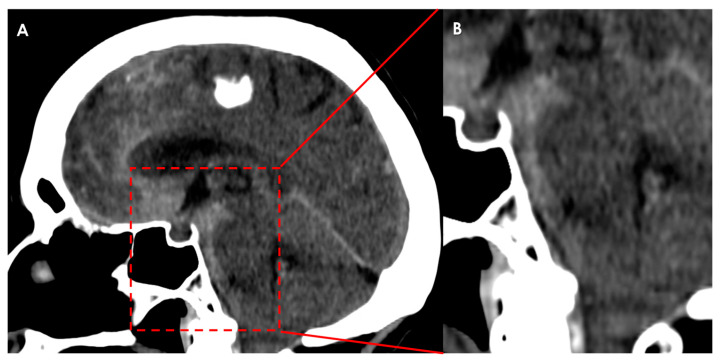
Non contrast CT of the brain (128 rows scanner, slice thickness 3 mm) in a 45-year-old patient with spontaneous non-aneurysmal SAH, reconstructed with multiplanar reconstruction protocol in sagittal plane (**A**) and magnified detail (**B**). In both panels, a tiny grayish linear structure is made evident in the prepontine cistern by the natural contrast of the extravasated blood, being highly suspicious for being SLYM.

Acute aneurysmal subarachnoid hemorrhage (aSAH) presents a unique opportunity to utilize blood as a natural contrast agent. It is hypothesized that the SLYM is impermeable to cellular blood components, which may allow for potential differentiation in imaging [141,142]. The proposed study aims to determine whether imaging techniques can be utilized to evaluate the compartmentalization of the subarachnoid space and indirectly infer the anatomical presence of the SLYM by using acute aSAH as a model. This approach could enhance our understanding of the existence and role of the SLYM within the context of SAH and its potential impact on CSF flow dynamics and related pathological conditions. An example of the application of the methods described in this study [138] is presented in Figure 3.

This study [138] represents a pioneering in vivo investigation aimed at assessing the compartmentalization of the subarachnoid space by utilizing the distribution of blood products in the context of acute aneurysmal subarachnoid hemorrhage (aSAH). The findings suggest a potential imaging-based correlation with the recently identified SLYM, which divides the subarachnoid space into two distinct compartments. This correlation is particularly evident in lower-grade aSAH (MFS 1–2), where blood acts as an internal contrast agent, highlighting the suspected location of the SLYM. Conversely, cases with more extensive hemorrhage (MFS 3–4) present challenges in visualizing the partition layer. The difficulty in visualization may stem from membrane displacement or rupture due to the high blood volume and elevated pressure within these confined spaces.

Future research could enhance anatomical understanding by exploring the feasibility of measuring the distance between the suspected SLYM and adjacent structures, as well as quantifying the thickness of the blood layer. Although these metrics are currently limited by the heterogeneous distribution of blood and variability in cisternal dimensions, they hold the potential to provide more objective insights into subarachnoid compartmentalization.

Identifying the SLYM and understanding its role could have significant implications for various neurological disorders. For example, in neurodegenerative diseases like Alzheimer’s, where CSF flow is disrupted, the compartmentalizing function of the SLYM could help explain these abnormalities. Targeting the SLYM or its pathways might offer novel therapeutic opportunities.

The discovery of the SLYM could lead to the development of imaging-based tools to evaluate CSF dynamics and identify patients at risk for disorders such as normal pressure hydrocephalus (NPH), thereby guiding treatment decisions.

A summary of the features of the SLYM is provided in the following table (Table 2).

### 4.3. Arachnoid Cysts

Arachnoid cysts are frequently encountered in imaging studies, accounting for approximately 1% of non-traumatic intracranial space-occupying lesions [143,144]. First described by Bright in 1831 [145], these cysts can be classified as either developmental (the majority) or familial, and they typically present in the first two decades of life, predominantly during the neonatal period. Males are affected in over two-thirds of cases [143,146].

On neuroimaging studies, arachnoid cysts appear as well-circumscribed, hypodense, non-enhancing mass lesions associated with the subarachnoid cisterns [147,148]. They contain clear, colorless fluid that resembles normal CSF [149]. In a study by Rengachary and Watanabe reviewing 208 cases, the majority of cysts were located in specific areas:

- Sylvian fissure: 103 (49%);
- Cerebellopontine angle: 22 (11%);
- Supracollicular area: 21 (10%);
- Vermian area: 19 (9%);
- Sellar and suprasellar regions: 18 (9%);
- Interhemispheric fissure: 10 (5%);
- Cerebral convexity: 9 (4%);
- Clival region: 6 (3%).

Many cases described as over the cerebral convexity actually originate from adjacent fissures and extend into the convexity. Bilateral temporal arachnoid cysts have been associated with conditions such as glutaric aciduria type 1 [150,151], tuberous sclerosis [152], and neurofibromatosis [153]. The clinical manifestations of arachnoid cysts depend on their location and the degree of neural compression caused by their expansion.

The Ball Valve Hypothesis proposes that a one-way valve between the cyst and the subarachnoid space facilitates cyst expansion. The Osmotic Gradient Hypothesis suggests that differences in osmotic pressure between the cyst contents and CSF contribute to expansion. Additionally, some theories indicate that cells lining the cyst walls may produce fluid. However, the first and last theories are challenged by observations of certain cysts that remain static, regress, or disappear altogether [143].

Rengachary and Watanabe described the microscopic features of arachnoid cysts, noting that:

- The subarachnoid space may narrow and potentially obliterate when traced from a normal area toward the cyst.
- At the cyst margin, the arachnoid membrane splits, enclosing the cyst.
- The outer wall contains dense connective tissue with compacted collagen fibers, while the inner wall has loosely arranged collagen fibers.
- The cyst cavity is clear, devoid of proteinaceous material or arachnoid trabeculae.

These findings support the notion that arachnoid cysts are intra-arachnoid and likely form from the splitting or duplication of the arachnoid membrane [149].

Historical theories of pathogenesis [145,154,155,156,157] are:

- Bright’s Theory: Attributed the pathogenesis of arachnoid cysts to the anomalous splitting of the arachnoid membrane.
- Starkman’s Theory: Proposed that small aberrations in CSF pulsation and flow during development could lead to the sequestration of a chamber within the arachnoid membrane.
- Robinson’s Theory: Suggested that differences in hemispheric volumes due to agenesis could result in CSF accumulation and cyst development. However, this theory was later rejected by Shaw [158] and retracted by Robinson in favor of Starkman’s theory.
- Head Trauma: Choi and Kim, along with other authors, reported that head trauma in infancy may contribute to the pathogenesis of arachnoid cysts. They noted a latent period between head trauma and the initial clinical manifestation ranging from 10 months to 6.2 years, with a mean of 2.2 years [159,160].

**Figure 4:**
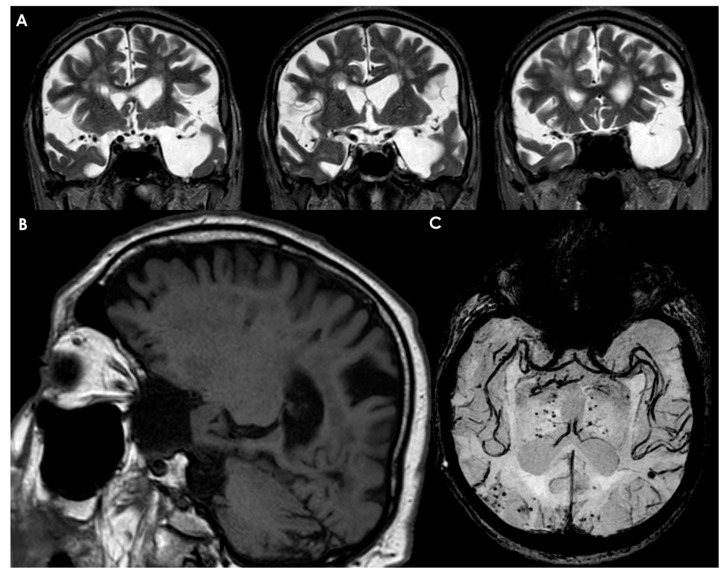
Brain MRI (1.5 T) of a 85 years old patient with a diffuse cerebral atrophy and a left temporo-polar arachnoid cyst (coronal T2W in panel (**A**), sagittal T1W in panel (**B**) and Susceptibility Weighted Imaging reconstructed according with Minimum Intensity Projection and MPR protocols in panel (**C**)). Middle cerebral artery on both sides is coursing within an enlarged sylvian cistern and close to a CSF-filled temporo-polas arachnoid cyst on the left side.

An example is illustrated in Figure 4.

Based on findings from CT scans and CT cisternography examinations, Galassi et al. [161,162,163] classified arachnoid cysts located in the middle cranial fossa into three distinct types: Type I, Type II, and Type III.

Type I is a mild form with the following features:

- Small, spindle-shaped lesion confined to the anterior temporal fossa.
- Compresses the anterior temporal pole posteriorly.
- Does not affect the ventricles or midline structures.
- Demonstrates free communication with the subarachnoid space and basal cisterns.
- Generally associated with mild symptoms due to limited compression on surrounding brain structures.

Type II is the classical type with the following characteristics:

- Medium-sized lesion, roughly triangular or quadrangular in shape.
- Occupies the anterior and middle parts of the temporal fossa, leading to a shortened temporal lobe.
- Extends into the Sylvian fissure, resulting in a widely opened fissure with the insula exposed.
- Communication with the subarachnoid space and cisterns is present but appears less pronounced than in Type I.
- Clinical Implications: Can lead to moderate symptoms due to increased compression on the temporal lobe and adjacent structures.

Type III is a severe form with the following characteristics:

- Large, round or oval-shaped lesion occupying nearly the entire temporal fossa and a significant area of the cerebral hemisphere.
- Results in atrophy of the temporal lobe and severe compression of the frontal and parietal lobes.
- Involvement of the ventricles and midline structures is observed.
- Unlike the first two types, Type III does not exhibit communication with the subarachnoid space and cisterns.
- Clinical Implications: Often associated with severe neurological deficits due to extensive compression and structural changes in the brain.

The varying types of arachnoid cysts lead to different clinical presentations primarily based on the degree of compression exerted on brain tissue. The classification is crucial for determining appropriate management plans, including observation, surgical intervention, or further imaging studies. The classification may also reflect different developmental stages of the arachnoid cysts, as previously suggested by Rengachary and Watanabe [161,162,163].

### 4.4. Perivascular Spaces

Perivascular spaces (PVS) are fluid-filled regions surrounding small blood vessels in the brain and function similarly to lymphatic vessels by facilitating the clearance of metabolic waste. When these spaces exceed 1 mm in diameter, they are classified as enlarged perivascular spaces (EPVS). While previously considered benign, emerging evidence suggests that EPVS may be associated with various clinical conditions. PVS were first described in the 19th century by Durand-Fardel and Pestalozzi, but their functional implications have only recently been clarified [164].

Perivascular spaces, also known as Virchow-Robin spaces, encase perforating cerebral arteries. Initial research focused on their anatomical and pathological characteristics, but advancements in MRI technology have led to increased attention on PVS and numerous clinical studies. As transportation channels for CSF, PVS may play a role in cerebrovascular, neurodegenerative, and neuroinflammatory diseases [165,166,167].

EPVS are particularly prevalent in the aging population, with advanced age identified as a significant risk factor. Studies indicate a correlation between EPVS and increased risks of cognitive decline, dementia, stroke, and cerebral small vessel disease (CSVD) [168,169,170]. Although the mechanisms underlying this correlation remain unclear, factors such as vascular stiffness, brain atrophy, and metabolic substance deposition may contribute to the development of EPVS and associated conditions [171].

Understanding the anatomical structure of perivascular spaces has evolved over time. Initially thought to connect directly to the subarachnoid space, it was later discovered that PVS surrounding cerebral cortical arteries are enclosed by vascular walls and a monolayer of pial meninges [171]. High-resolution MRI has enhanced our understanding of perivascular space anatomy, revealing that their structure varies depending on their location, such as in the basal ganglia and cerebral hemispheres [104].

EPVS is believed to play a role in the peripheral circulation of the central nervous system, facilitating the transport of CSF and the removal of metabolic waste. The primary components of PVS include interstitial fluid, macrophages, and various proteins, such as amyloid beta and immunoglobulin G [172]. This process involves the distribution of CSF through periarterial spaces and the exchange of CSF with interstitial fluid, ultimately aiding clearance through meningeal lymphatics [173].

Research suggests that EPVS in aging populations is linked to complex mechanisms, including arteriolosclerosis and the deposition of amyloid beta and tau proteins, which may be associated with neurodegenerative diseases [112]. Animal studies indicate that older mice exhibit decreased vessel pulsatility and reduced amyloid clearance compared to younger mice [174]. Furthermore, inflammatory processes mediated by microglia may connect glymphatic dysfunction and Alzheimer’s disease, suggesting that anti-neuroinflammatory therapies could mitigate the effects of EPVS [174]. A community-based study found that EPVS is associated with infarcts, independent of other neuropathologies, indicating that PVS may share neurobiological pathways with these infarcts [175]. However, current research on EPVS and age-related pathologies remains preliminary and warrants further investigation.

Several causal mechanisms for the enlargement of EPVS have been proposed, including:

- Arterial Stiffening: Aging leads to arterial stiffening, which contributes to EPVS enlargement due to vascular wall damage and remodeling. High pulse waves may exacerbate the vulnerability of perforating arteries in the basal ganglia region [168,176].
- Protein Aggregation: Abnormal protein aggregation, such as amyloid beta, may obstruct upstream cortical arteries, leading to interstitial fluid drainage issues and subsequent EPVS expansion [174].
- Brain Atrophy: Age-related brain atrophy creates a cavitation effect, pulling on tissue surrounding blood vessels and resulting in EPVS dilation [177].
- Blood–Brain Barrier Dysfunction: Damage to the blood–brain barrier (BBB) due to factors such as high blood pressure can increase BBB permeability, resulting in fluid leakage and EPVS enlargement [177].

These proposed mechanisms require further validation through concrete evidence.

Risk factors for EPVS include demographic characteristics, genetics, hypertension, and sleep duration. Advanced age correlates significantly with the severity of EPVS, and research indicates that men may experience a higher prevalence than women [170]. Additionally, ethnic differences have been observed, with lower EPVS prevalence reported among Chinese patients compared to white patients with transient ischemic attack (TIA) or ischemic stroke [170].

Vascular risk factors, particularly hypertension, play a crucial role in the development of EPVS. Chronic stress and inflammation can lead to vascular damage, promoting EPVS enlargement. Studies have demonstrated that systolic blood pressure is independently correlated with EPVS in the basal ganglia, underscoring the importance of blood pressure management [178].

Sleep quality also affects EPVS, with poor sleep efficiency linked to increased basal ganglia perivascular spaces. Research suggests that deep sleep enhances the metabolism of waste substances, indicating that sleep disturbances could be a risk factor for EPVS expansion [179].

EPVS has been identified as a potential marker of cognitive decline and dementia. Research indicates a significant association between EPVS and cognitive impairment, independent of other CSVD markers [175]. Studies show that higher counts of EPVS correlate negatively with cognitive assessment scores, highlighting a connection between EPVS and dementia risk [170].

EPVS are increasingly recognized as important markers in the assessment of cerebral small vessel disease (CSVD) and its associated pathologies. Their patterns can provide insights into differentiating between conditions such as cerebral amyloid angiopathy (CAA) and hypertensive arteriopathy, particularly in cases of spontaneous intracerebral hemorrhage. High degrees of EPVS in the basal ganglia are correlated with hypertensive intracerebral hemorrhage. This suggests that chronic hypertension leads to changes that manifest as EPVS in this region. Conversely, EPVS located around the ventricles is associated with CAA-related hemorrhage. This distinction is crucial for understanding the underlying pathology in patients presenting with intracerebral hemorrhages.

The interaction between EPVS and other markers of CSVD is complex, and their roles in cognitive impairment remain subjects of debate. It is believed that conditions such as arteriolosclerosis may lead to increased cortical arterial pulsatility or hypoperfusion, contributing to the development of EPVS, white matter lesions, and lacunae [180]. The clinical effects of EPVS, white matter lesions, and lacunae may not be identical, even though they can arise from similar pathophysiological processes [181,182,183].

Some studies suggest that the relationship between EPVS and cognitive decline, particularly in relation to the presence of lacunae, may be overestimated [184,185]. This indicates a need for further research to clarify the specific contributions of each marker to cognitive impairment.

In summary, EPVS is closely associated with various clinical diseases and serves as a promising potential indicator of cognitive impairment, disease progression, and prognosis. However, significant heterogeneity exists among research methods. Therefore, future studies should aim for unification and standardization of research methodologies. Additionally, some previous studies did not investigate the role of other CSVD in their evaluation of EPVS; thus, more systematic work is needed to further confirm these conclusions.

#### Physiological Background of MRI Evaluation of Perivascular Spaces

As previously mentioned, PVS, also known as Virchow-Robin spaces, are fluid-filled compartments that surround cerebral blood vessels as they penetrate the brain parenchyma. Their significance has gained increasing attention in neuroimaging studies, especially with the advent of advanced MRI techniques that enable detailed visualization of these structures [167].

PVS are characterized as fluid-filled spaces enveloping cerebral arteries and veins. They are externally demarcated by the glia limitans, a structure formed by the endfeet of astrocytes and an outer basal lamina. PVS are critical components of the brain’s clearance system, facilitating the exchange of CSF and interstitial fluid (ISF), and playing a vital role in the brain’s waste clearance mechanisms [56].

The glymphatic system, a term introduced in 2012, describes the process through which CSF flows into PVS and mixes with ISF, promoting the clearance of metabolic waste products from the brain [56]. This process is driven by arterial pulsations and is essential for maintaining homeostasis within the CNS. Recent advancements in imaging techniques have enabled the exploration of PVS dynamics and their alterations in various neurological conditions [57].

**Figure 5:**
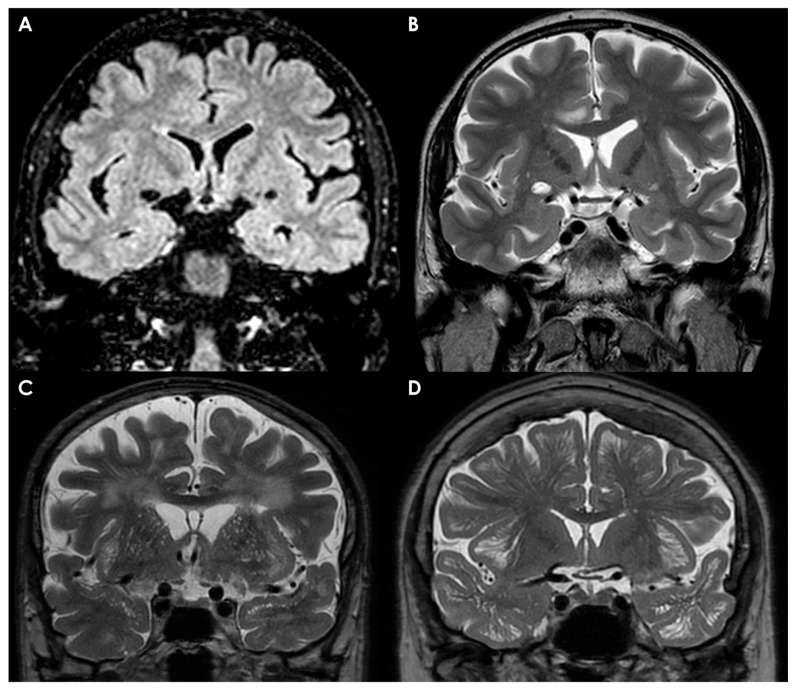
Brain MRI of different subtypes of EPVSs. In panel (**A**) (coronal FLAIR) and (**B**) (coronal T2), a large PVS in the right basal ganglia location (under the lenticular nucleus) is evident. In panels (**C**,**D**), there are two different examples in coronal T2, showing multiple deep (panel (**C**)) and lobar (panel (**D**)) EPVSs.

MRI has become the gold standard for visualizing PVS due to its high sensitivity to the properties of cerebrospinal fluid. On MRI, PVS appear as tubular, fluid-filled structures, particularly prominent in regions such as the basal ganglia and the centrum semiovale [167]. The signal intensity of PVS on T1-weighted images is generally low, while they appear hyperintense on T2-weighted images, indicating that the fluid within these spaces is similar to that of CSF. An example is proposed in Figure 5.

Visual rating scales are commonly used to assess PVS on MRI. Various scoring systems have been developed, allowing for the classification of PVS based on their count and size in specific brain regions [186,187]. While these systems are relatively straightforward, they often lack the granularity needed for precise assessments, particularly when PVS counts are near threshold limits separating different categories [188].

Recent advancements in imaging technology have led to the development of automated and semi-automated methods for PVS segmentation. These techniques utilize various filtering and thresholding methods to enhance PVS visibility and quantify their morphological features [189]. Metrics such as PVS volume, cross-sectional diameter, and linearity can now be computed, providing valuable insights into PVS characteristics and their correlation with neurological conditions [190].

With the increasing availability of high-resolution neuroimaging datasets, machine learning and deep learning methods have been employed for PVS segmentation. These data-driven approaches have demonstrated enhanced accuracy compared to traditional methods, allowing for improved analysis of PVS morphology and distribution [191].

Despite the progress in PVS imaging, challenges remain in differentiating PVS from other lesions, such as white matter hyperintensities (WMH) and lacunes [180]. Understanding the anatomical distribution and typical MRI characteristics of PVS is crucial for accurate identification. Additionally, enhancing PVS contrast through advanced imaging techniques and post-processing methods can aid in improving visualization and quantification [192].

Understanding the fluid dynamics within PVS is essential for elucidating their physiological roles. Recent studies have shown that arterial pulsations drive fluid movement in PVS, with net flow direction mirroring blood flow [193]. Factors such as respiratory movements and the sleep–wake cycle also influence PVS fluid dynamics [194]. Advanced MRI techniques, including phase-contrast imaging, are being explored to assess CSF and blood flow dynamics noninvasively [195].

Diffusion MRI (dMRI) is a valuable tool for studying PVS fluid dynamics. Techniques such as diffusion tensor imaging (DTI) can provide insights into water diffusion patterns within PVS and surrounding tissues [196,197]. By employing multi-compartment modeling approaches, researchers can isolate the contributions of PVS to diffusion metrics, enhancing our understanding of their functional implications in health and disease [196,197].

Technological advancements have facilitated the study of PVS structure across different age groups. Research indicates that the number and volume of MRI-visible PVS increase with age, particularly in the elderly population [198,199]. This age-related enlargement may reflect the physiological changes associated with aging and has implications for understanding cognitive decline [200].

## 5. Conclusions

Understanding the leptomeninges, which include the arachnoid and pia mater, is crucial for the protection of the CNS and the proper functioning of CSF circulation. This study highlights the importance of these meningeal layers not only as protective barriers but also as key players in brain development and function. Their roles in CSF dynamics and neurovascular interactions have significant implications for various neurological conditions, including hydrocephalus and neurodevelopmental disorders. Future research should focus on elucidating the cellular properties and functional dynamics of the leptomeninges, as this knowledge may lead to improved diagnostic and therapeutic strategies for managing disorders that disrupt CSF flow and brain health. In addition, future research should explore the cellular properties of the perivascular arachnoid barrier and its potential role as an immune interface, as neuroinflammatory conditions could alter the immune cell content in the subarachnoid space, affecting solute transport. In summary, our study provides compelling evidence for compartmentalized CSF flow in the subarachnoid space, emphasizing the importance of the perivascular subarachnoid space in solute transport and its implications for neurological health and disease.

## Figures and Tables

**Table 1:** Key aspects of cerebrospinal fluid CSF flow.

Aspect	Description
CSF Production	Primarily produced by the choroid plexus in the ventricles at a rate of approximately 20 mL/h.
CSF Volume	Total volume in adults is about 130 mL, distributed as follows:
	- Ventricles: approximately 30 mL
	- Subarachnoid space around the brain: around 25 mL
	- Subarachnoid space around the spinal cord: approximately 75 mL
Flow Pathway	1. CSF flows from the choroid plexus into the ventricles.
	2. Exits the ventricles via the foramina of Luschka and Magendie into the subarachnoid space.
	3. Moves through the basal cisterns and over the brain’s convexities, flowing down the spinal cord.
Driving Forces	- Hydrostatic pressure gradients from the choroid plexus to arachnoid villi.
	- Pulsations from the choroid plexus, respiratory, and cardiac rhythms contributing to pulsatile flow.
Clearance Mechanisms	- Absorption into the blood of the cerebral venous sinuses through arachnoid villi.
	- Involves drainage pathways to cervical and spinal lymph nodes.
Glymphatic System	- CSF enters perivascular spaces, mixing with interstitial fluid (ISF) for waste clearance.
	- Movement driven by arterial pulsations and astrocytic endfeet enriched with aquaporin-4 (AQP4).
Pathological Implications	- Impairments in CSF dynamics linked to conditions such as idiopathic normal pressure hydrocephalus (iNPH) and neurodegenerative diseases.

**Table 2:** Main roles of the SLYM.

Role	Functions
SLYM Structure	Outer Superficial Compartment: Contains CSF and is involved in waste clearance. Inner Deep Compartment: Lines the brain and restricts the movement of larger molecules.
Functions of SLYM	Compartmentalization: Divides the subarachnoid space into two distinct compartments, facilitating CSF dynamics. Barrier Function: Limits the passage of molecules greater than 3 kDa, including neurotoxic substances like amyloid-β and tau. Fluid Regulation: Facilitates the movement of CSF and ISF for nutrient delivery and waste removal.
Pathophysiological Implications	Impact on CSF Dynamics: Disruptions in the SLYM can lead to conditions such as NPH and other neurodegenerative diseases. Role in Immune Surveillance: The SLYM may also serve as a barrier for immune cell migration, affecting the brain’s response to injury and disease.
Imaging and Research Techniques	In Vivo Imaging: Utilizes techniques like two-photon microscopy to visualize the SLYM and assess its role in CSF dynamics. Contrast Agents: Challenges in using traditional contrast agents (e.g., gadolinium) due to size limitations but using blood as a natural contrast agent in acute aSAH.
Clinical Significance	Potential for Therapeutic Targets: Understanding the SLYM’s function may lead to novel treatment options for various neurological disorders.

## Data Availability

No new data were created or analyzed in this study. Data sharing is not applicable to this article.
